# Exploring the relationship between sunlight exposure, psychological health, and gestational weight gain: a prospective observational study

**DOI:** 10.1186/s12889-024-17677-w

**Published:** 2024-01-09

**Authors:** Satvinder Kaur, Ee Yin Kok, Nor Aini Jamil, Susy K. Sebayang

**Affiliations:** 1 Department of Food Science and Nutrition, Faculty of Applied Sciences, UCSI University, 56000 Cheras, Kuala Lumpur Malaysia; 2 Centre for Community Health Studies (ReaCH), Faculty of Health Sciences, Universiti Kebangsaan Malaysia, 50300 Kuala Lumpur, Malaysia; 3 Research Group for Health and Well-Being of Women and Children, Universitas Airlangga, Surabaya, Indonesia; 4 Faculty of Health, Medicine and Life Sciences, Universitas Airlangga Banyuwangi Campus, Jalan Wijaya Kusuma No 113, Banyuwangi, East Java, 68425 Indonesia

**Keywords:** Gestational weight gain, Depression, Pregnant women, Sleep quality, Sunlight exposure, Maternal health

## Abstract

**Introduction:**

Gestational weight gain (GWG) is influenced by various factors during pregnancy. This study attempts to explore the relationship between environmental factors i.e., sunlight exposure and psychological health i.e. psychological well-being and sleep quality during pregnancy with total gestational weight gain.

**Methods:**

This was a prospective observational study conducted in government maternity clinics in Kuala Lumpur. Pregnant women aged 19–39 years without comorbidities were recruited during second trimester and followed up until birth. The participants were required to wear a UVB dosimeter for a total of three consecutive days (2 weekdays and 1 weekend) to determine sunlight exposure (SED) during their second trimester. The PSQI and DASS-21 were used to determine sleep quality and psychological wellbeing, respectively. GWG data were collected from clinic health records. The association of sun exposure and psychological health with total GWG was determined using multiple linear regression.

**Results:**

A total of 73 pregnant women aged 27.9 ± 3.3 years were included in the analysis. The prevalence of pregnant women exhibiting stress, anxiety, and depression symptoms was 11%, 40%, and 16% respectively. The global PSQI median score was 5 (IQR = 3), with 59% having poor sleep quality. Median sleep duration was 7 h (IQR = 2) while median sleep efficiency was 92% (IQR = 14). The median SED was 0.04 (IQR = 0.09), with 51% of them being under the 50th percentile. The majority had adequate GWG (58%). Sleep parameters were not found to be correlated with total GWG except for sleep latency (*ρ* = -0.356, *p* = 0.002). Sunlight exposure was found to have no significant relationship with sleep and total GWG. Adjusted multiple linear regression showed that greater depression is associated with higher total GWG (β = 0.239, *p* = 0.039) while controlling for sleep quality.

**Conclusion:**

Depression was associated with total GWG when sleep quality was controlled for while sunlight exposure had no significant association with GWG. Future studies should study the complex relationship between factors of mental health, sleep, and weight gain during pregnancy. Healthcare providers may be better equipped to develop interventions aimed to prevent negative maternal and fetal health outcomes.

## Introduction

In Malaysia, excessive gestational weight gain is a significant issue, with prevalence ranging from 20–40% in individual studies, indicating the need to study factors associated with excessive GWG [1, 2]. Excessive GWG has been associated with negative health implications for both the mother and the baby, such as increased risks of gestational diabetes mellitus, delivery complications, and macrosomia [3]. While excessive GWG is seen as an emerging trend, the other end of the spectrum, which is inadequate GWG is also as important. Several studies including a meta-analysis found that the prevalence of inadequate GWG is rising (20–40%) especially in low and middle income countries [4, 5]. Both normal and underweight Southeast Asia women were found to not adhere to the minimum requirement of weight gain during pregnancy [5]. High burden of inadequate weight gain should be highlighted in the region especially among normal weight pregnant women.

GWG is an important indicator of proper fetal growth, which later minimizes the risk of pregnancy and birth complications and promotes postpartum health for both the mother and the infant [6]. During pregnancy, several changes impact women’s gestational weight gain (GWG) either directly or indirectly. Direct factors that influence maternal weight gain include women’s age, pre-pregnancy weight, the number of fetus, and underlying health conditions [6]. Meanwhile, indirect factors include psychological well-being, which influences dietary intake, and environmental exposure that can impact maternal metabolism [7–9]. Another factor for healthy GWG is sleep quality. Good sleep quality during pregnancy is found to be associated with fetal development and maternal mood [7, 10].

Environmental exposures such as bright light, and pollution were all found to be associated with epigenetic changes in adiposity leading to higher GWG [8, 9, 11]. At current times, more studies are required to determine the role of sunlight exposure to pregnancy outcomes as some past studies showed a positive effect on asthma when pregnant women received proper sunlight exposure in the second trimester [12].

Maternal psychological health, which refers to a woman’s mental and emotional well-being during pregnancy, has been found to play a crucial role in GWG through epigenetic changes [13]. Increased levels of stress, anxiety, and depression during pregnancy have been linked to unfavorable GWG and positive psychological health has been linked with healthier GWG [14]. The mechanism related to this was found to be interplayed with maternal sleep quality and dietary choices. Depressive symptoms, for example, often result in poorer diet choices and lead to an unhealthy eating pattern [15]. While it is clearly established that diet plays a direct role in this association, lesser is known about sleep and environmental factors.

Sunlight exposure plays a significant role in regulating the human sleep–wake cycle, known as the biological clock or circadian rhythm. Exposure to natural sunlight during the day aids to synchronize the internal clock with the external environment. The suprachiasmatic nucleus (SCN) is in the hypothalamus of the brain and is often known as the “internal and master clock” of the body that regulates physiology and behavior [16]. The sleep–wake cycle regulation happens via light exposure detected at the eye level and this in turn promotes the production of melatonin, a hormone that is required for sleep induction, that is secreted from the SCN cue. Bright light exposure, especially in the morning, helps in the process of internal clock regulation and promotes alertness during the day [17].

Sunlight exposure during pregnancy has been suggested to play a role in GWG through the production of vitamin D, especially in second trimester [11]. Optimum levels of vitamin D were maintained via adequate sunlight exposure as the ultraviolet rays from sunlight produce vitamin D when skin is exposed to sunlight. Pregnant women with vitamin D insufficiency had both issues with excessive and inadequate weight gain [18]. Other means of sunlight exposure influencing GWG is via metabolic health. Higher sunlight exposure was found to lower insulin resistance, which in turn was associated with lower GWG [19]. Besides that, sunshine enhances happiness and subsequently programs a positive well-being, hence, exposure to more sunlight throughout pregnancy offers measures to combat stressful events [11].

Given the intricacy of the link between sunlight exposure, psychological wellbeing and GWG, and the scarcity of publication in this area, studying the association of all these factors during pregnancy is crucial. This study, thus, aims to assess the relationship of sunlight exposure and psychological wellbeing with total GWG among second trimester pregnant women in Kuala Lumpur, Malaysia.

## Methodology

### Study design and participants

**Fig. 1:**
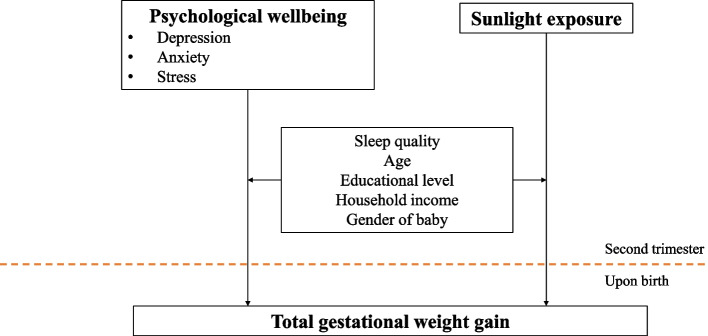
Conceptual framework of the study

This was a prospective observational study where pregnant women were recruited in the 2nd trimester to collect baseline maternal data and followed up after they have given birth to obtain their total GWG. Pregnant women were recruited through convenience sampling from nine government maternity clinics in Kuala Lumpur that were selected through simple random sampling. Data collection was conducted from July 2019 to April 2021. Malaysian primigravidas who were aged 19–39 years old, literate in English or Bahasa Melayu, and having singleton pregnancy were recruited for this study. Pregnant women who were diagnosed with gestational diabetes mellitus, hypertension, or anaemia were excluded from the study. A conceptual framework of our study was depicted in Fig. 1. All procedures adhered to the ethical standards outlined in the Declaration of Helsinki. The Medical Research and Ethics Committee (KKM/NIHSEC/P19-125) and the National Medical Research Registrar (NMRR-18–3412-45,225) approved all experimental protocols, ensuring compliance with the committee's ethical guidelines. Prior to data collection in government maternal clinics, approvals were obtained from Health Department of Kuala Lumpur and Putrajaya. Besides, a written informed consent form was collected from the participants prior to data collection.

Recruitment and data collection were done by research enumerators at the maternity clinics. All research enumerators were trained prior to collecting data to ensure data quality. Collected responses were cross checked by research team to screen for data validation, data verification and missing data.

#### Sample size

Based on the probability of poor sleep quality among pregnant women with adequate GWG (*P* = 0.33) and inadequate or excessive GWG (*P* = 0.67), confidence level at 95% and 80% power, the minimum sample size required was 40 participants [20]. After including 50% of dropout rate, a minimum of 60 participants should be recruited for the study.

### Measures

#### Sunlight exposure

A polysulphone film dosimeter (UV dosimeter) was used to determine natural UVB exposure. Each subject received one polysulphone film badge to be worn on the clothing for three consecutive days (two weekdays and one weekend day). The badge was stored in a sealed envelope provided when it is not worn. Analysis of the badges was done using V-1200 Spectrophotometer, Leuven, Belgium, in Universiti Kebangsaan Malaysia laboratory, where absorbance was measured at 330 nm wavelength. The change in absorbance was calculated using the formula below to compute UVB dose.

$$\mathrm{SED }= 10.7 (\mathrm{\Delta A}330) + 14.3 (\mathrm{\Delta A}330)2 - 26.4 (\mathrm{\Delta A}330)3+ 89.1 (\mathrm{\Delta A}330)4;$$

where ΔA330 is the change in absorbance of the film badge pre- and post-UVB exposure. Sunlight exposure was ranked according to upper 50th and lower 50th percentiles.

#### Psychological well-being

Depression, Anxiety, and Stress Scale – 21 items (DASS-21) was used to assess the levels of depression, anxiety, and stress of pregnant women over the past week [21]. The questionnaire consists of 7 items for each component: depression, anxiety, and stress. Participants rated their condition according to a 4-point Likert scale of 0 (did not apply to me at all) to 3 (applied to me very much or most of the time) for the past week. The scores for each subscale were then summed up and multiplied by 2 to get the final score. The participant’s psychological well-being was categorized based on the severity of each component. The cut-off values for depression were normal (0–9), mild (10–13), moderate (14–20), and severe (≥ 21). For anxiety symptoms, the corresponding cut-off values were normal (0–7), mild (8–9), moderate (10–14), and severe (≥ 15). The suggested cut-off scores for stress symptoms were normal (0–14), mild (15–18), moderate (19–25) and severe (≥ 26).

#### Sleep quality

The sleep quality of pregnant women was measured using the modified Pittsburgh Sleep Quality Index (PSQI), which evaluated the sleep quality for the past month, with additional questions to differentiate sleep time and wake time between workdays and work-free days [22]. PSQI is a validated questionnaire that measures seven derived components of sleep quality: subjective sleep quality, sleep latency, habitual sleep efficiency (%), sleep duration (hours), sleep disturbances, use of sleep medication, and daytime dysfunction. Each subdomain was scored from 0 to 3, where a higher value corresponds to more severe sleep difficulties. The summed scores from all subdomains were produced to calculate the total global PSQI score, ranging from 0 to 21. A total global score of more than 5 indicated poor sleep quality, and vice versa. A Malay version of the PSQI was also used in the current study, it has been tested reliable from past studies with Cronbach’s alpha of 0.74 and test–retest reliability (ICC) of 0.58 [23].

#### Gestational weight gain

Data on GWG of the participants were collected through the maternity health records by calculating the difference between the maternal weight at their last check-up and pre-pregnancy weight which was recorded during their first antenatal appointment. Their weight was measured by trained nurses from the facilities during their antenatal health check. The total GWG was compared with the Institute of Medicine (IOM) guidelines (2009) for recommended GWG to determine adequacy of GWG. For pregnant women with normal BMI prior to pregnancy, they are recommended to gain 11.5 to 16 kg throughout their pregnancy period, weight gain will be classified as inadequate or excessive if it is not within the recommended range.

### Statistical analyses

Data cleaning was conducted to identify and correct inconsistencies in data. This includes removing duplicate data, fixing typos, and standardizing data format. Statistical analysis was performed using SPSS version 23 software (IBM, Chicago, USA). Normality of data distribution was determined using the Shapiro Wilk test. Descriptive statistics were presented as mean (SD) for normally distributed data, and median (IQR) for non-normally distributed data. Correlation analysis was conducted to investigate the relationship between sunlight exposure, psychological well-being, and sleep quality with total GWG. Next, the association between sunlight exposure and psychological well-being during pregnancy with total GWG was evaluated using multiple linear regression. Sleep quality, age, educational level, monthly household income, and gender of baby were adjusted for in the analysis. Observed association was expressed as beta coefficient (β) corresponding to 95% confidence intervals (CI). A p-value of more than 0.05 was considered statistically significant.

## Results

A total of 92 pregnant women were recruited from government maternity clinics in Kuala Lumpur. There was a 20% dropout rate upon delivery and 1 participant did not complete the PSQI. Finally, a total of 73 primigravids participated in the study. From Table 1, it was reported that the majority of the pregnant women had tertiary educational level (82%) and from middle household income families (62%). More than half (65%) of the pregnant women had normal BMI, while the remaining were underweight (11%), overweight (19%) and obese (6%). In terms of sleep quality, the median PSQI score was 5 (IQR = 3), with 41% of the pregnant women having poor sleep quality (PSQI score ≥ 5). Median sleep duration was 7 h (IQR = 2) while sleep efficiency was 92% (IQR = 14). The average total GWG was 12.9 ± 5.2 kg. A total of 58% of the pregnant women had adequate GWG, while 30% of them had inadequate weight gain, and 12% had excessive weight gain according to the IOM guidelines. The median SED level was 0.04 (IQR = 0.09).

**Table 1:** Descriptive characteristics of study population (*N* = 73)

Variables	Median (IQR)	N (%)
**Age (years)**^**a**^	27.9 (3.3)	
**Educational level**		
Secondary		13 (17.8)
Tertiary		60 (82.2)
**Household income**^**b**^		
Low (< RM2300)		9 (12.3)
Middle (RM2300 – 5599)		45 (61.6)
High (> RM5599)		19 (26.0)
**Pre-pregnancy BMI (kg/m**^**2**^**)**^**a**^	23.20 (4.30)	
Underweight		8 (11.0)
Normal		47 (64.4)
Overweight		14 (19.2)
Obese		4 (5.5)
**Global PSQI score (0–21)**	5 (3)	
Poor sleep quality (≥ 5)		30 (41.1)
Good sleep quality (< 5)		43 (58.9)
Subjective sleep quality score (0–3)	1 (0)	
Sleep latency score (0–3)	1 (2)	
Sleep duration (hr)	7 (2)	
Sleep efficiency (%)	92 (14)	
Sleep disturbance score (0–3)	1 (1)	
Sleep medication score (0–3)	0	
Daytime dysfunction score (0–3)	1 (1)	
**Total Gestational Weight Gain (kg)** ^**a**^	12.9 (5.2)	
Adequate		42 (57.5)
Inadequate		22 (30.1)
Excessive		9 (12.3)
**Sunlight exposure**	0.04 (0.09)	
≤ 50th percentile		36 (49.3)
> 50th percentile		37 (50.7)

*Abbreviations*: *IQR* Interquartile Range, *RM* Ringgit Malaysia, *BMI* Body Mass Index, *PSQI* Pittsburgh Sleep Quality Index

^a^Reported in mean (SD)

^b^Based on 10th Malayisa Economic Plan

From Table 2, descriptive statistics of the psychological well-being for the pregnant women in this study was reported. The median score for stress among the pregnant women was 6 (IQR = 8), which indicates the pregnant women had normal stress levels on average (below 14). However, 5.5% and 1.4% of women had moderate and severe stress levels respectively. Notably, anxiety levels were relatively higher with 23.2% women had moderate anxiety levels (scored 10–14) and 11.0% of the pregnant women had severe anxiety levels (scored 15 and above). Nevertheless, the majority of the pregnant women (60.3%) had normal anxiety levels with median score of 6 (IQR = 6). While the median depression score was on the lower side with 4 (IQR = 6), and 83.6% of the pregnant women were on the normal level of depression, the prevalence of mild depression was 11% while severe depression was 2.7%.

**Table 2:** Descriptive statistics of psychological wellbeing (*N* = 73)

Variables	Median (IQR)	N (%)
**Stress**	6 (8)	
Normal (0–14)		65 (89.0)
Mild (15–18)		3 (4.1)
Moderate (19–25)		4 (5.5)
Severe (26–42)		1 (1.4)
**Anxiety**	6 (6)	
Normal (0–7)		44 (60.3)
Mild (8–9)		4 (5.5)
Moderate (10–14)		17 (23.2)
Severe (15–42)		8 (11.0)
**Depression**	4 (6)	
Normal (0–9)		61 (83.6)
Mild (10–13)		8 (11.0)
Moderate (14–20)		2 (2.7)
Severe (21–42)		2 (2.7)

*Abbreviation*: *IQR* Interquartile Range

According to Table 3, when assessing the correlations between psychological well-being (depression, anxiety, and stress) and sunlight exposure with total GWG, no significant findings were found. However, when correlation analysis was conducted between sleep parameters with total GWG, sleep latency score was found to be negatively correlated with GWG (*ρ* = -0.356, *p* = 0.002). The PSQI uses a reverse scoring, indicating that longer sleep latency is correlated with higher total GWG among the pregnant women in this study. Subjective sleep quality, sleep efficiency, sleep disturbance, daytime dysfunction, and sleep quality (Global PSQI score) were not significantly correlated with total GWG.

**Table 3:** Correlations of psychological wellbeing, sunlight exposure, and sleep parameters with total GWG (*N* = 73)

	Total GWG	
	Spearman’s rho (ρ)	*p*-value
Psychological wellbeing		
Stress score	0.163	0.169
Anxiety score	0.132	0.264
Depression score	0.133	0.262
Sunlight exposure (SED)	-0.133	0.262
Sleep parameters		
Subjective sleep quality	-0.195	0.098
Sleep latency	-0.356	0.002^*^
Sleep efficiency	0.023	0.845
Sleep disturbance	0.079	0.508
Daytime dysfunction	-0.142	0.230
Global PSQI score	-0.154	0.194

*Abbreviations*: *GWG* Gestational weight gain, *SED* Standard erythemal dose, *PSQI* Pittsburgh Sleep Quality Index

^*^p < 0.05

Lastly, results of the multiple linear regression showed that only depression was positively associated with total GWG (β = 0.239, *p* = 0.039, 95%CI = 0.013–0.466) when sleep quality is included into the model alongside with other sociodemographic variables (age, educational level, monthly household income, and gender of baby) (Table 4). One point increase in depression score was associated with 0.24 kg increase in weight gain. On the other hand, stress, anxiety, and sunlight exposure did not show any significant associations with GWG.

**Table 4:** Factors associated with total gestational weight gain (*N* = 73)

	Total GWG (kg)	
	β (95% CI)	*p*-value
Stress^a^	0.130 (-0.045 – 0.304)	0.143
Anxiety^b^	0.060 (-0.174 – 0.295)	0.610
Depression^c^	0.239 (0.013 – 0.466)	0.039^*^
Sunlight exposure (SED)^d^	0.077 (-5.865 – 6.019)	0.979

Multiple linear regression adjusted for sleep quality, age, educational level, monthly household income, and gender of baby

*Abbreviations*: *GWG* Gestational weight gain, *CI* Confidence interval

^a^Model significant at *p* = 0.002, R = 0.513, R^2^:0.263, adjusted R^2^: 0.195

^b^Model significant at *p* = 0.005, R = 0.491, R^2^:0.241, adjusted R^2^: 0.171

^c^Model significant at *p* = 0.001, R = 0.536, R^2^:0.287, adjusted R^2^: 0.221

^d^Model significant at *p* = 0.006, R = 0.488, R^2^:0.238, adjusted R^2^: 0.168

## Discussion

The study found that sleep latency was negatively associated with GWG. In addition, depression was positively associated with GWG even after adjusting for sleep and other covariates.. The findings suggests that pregnant women in urban areas with singleton pregnancies who experience depression during the second trimester are more likely to gain more weight by the end of their pregnancy. Furthermore, the study indicated that sunlight exposure during pregnancy was lower than what was observed in previous research done among Malaysians [24].

Sunlight exposure among Malaysian pregnant women is relatively low as compared to pregnant women in India [25] due to lifestyle norms and preferences. This is similar to a study in the UK done among South Asians, whereby the preference to stay indoors contributed to the low sunlight exposure [26]. In addition, this study was conducted in between the movement restriction order, whereby there were periods of home confinement due to COVID-19 control measures. Moreover, sunlight exposure could vary due to several factors such as seasonal changes and individual preferences [27, 28]. Asians are more inclined to avoid bright sunlight exposure and prefer to stay indoors [27].

Sleep quality has been found to influence GWG among pregnant women in many previous studies [7, 10, 29]. Sleep during pregnancy is an important factor for the health and well-being of both the mother and the developing fetus. Sleep latency, which is the amount of time it takes to fall asleep, has been found to be associated with GWG in the current study. Specifically, research suggests that longer sleep latency, which is the time taken to fall asleep, is correlated with higher total GWG [29]. In healthy adults, inability to sleep or prolonged time taken to sleep was found to be associated with insulin sensitivity and increased cortisol levels [30].

We found a significant association between mid-pregnancy depression with GWG after controlling for sleep quality. This suggests that depression may be an important factor to consider in efforts to promote healthy GWG during pregnancy. The relationship between depression and GWG may be mediated by other factors, such as physical activity and dietary habits that was not included in this study. A study by Cattane et al*.* (2021) found that depression during pregnancy was associated with decreased physical activity and poorer dietary habits, which in turn were associated with higher GWG [31]. Addressing depression during mid-pregnancy may help to promote healthy GWG. Cognitive-behavioral intervention aimed at reducing depression during pregnancy was found to be effective and it could also help in improve lifestyle practices such as improved dietary intake and physical activity that translates to healthy gestational weight gain [32, 33]. Healthcare professionals dealing with pregnant women should be aware about the link between depression and its role in gestational weight gain. Consideration in screening for depression as part of prenatal assessment could influence the control of excessive weight gain [34]. It is suggested that maternal mental health in Malaysia requires immediate attention as the perinatal mental health scope appears to be lacking in qualified professionals, despite the rising cases [35].

There are some limitations to our study. First, we did not study the role of physical activity and dietary intake in gestational weight gain. These are main variables linked to energy expenditure and therefore, could offer insights to the pattern of weight gain during pregnancy. Second, it was not possible to standardize the use of calibrated weighing scales as the data was collected in government clinics, raising the issues of measurement error related to instrumentation. Also, maternal factors were recorded in the second trimester. It is recommended that sunlight exposure and factors influencing weight gain be studied in all three trimesters, to provide a comprehensive overview of maternal exposure during pregnancy. Understanding the role of sunlight exposure in GWG requires a more comprehensive approach such as keeping a sun exposure diary to capture time of day and duration of exposure. Convenience sampling method used in this study may not be generalizable to the whole population and could include selection biases. Lastly, a larger sample size would offer wider representation of the Malaysian pregnant women population.

## Conclusion

The finding of this study found that mid-pregnancy depression is associated with total gestational weight gain highlights the need for policies and programs that address maternal psychological well-being during pregnancy. Healthy gestational weight gain is essential for optimum maternal and infant health outcomes, therefore, understanding the link and mechanistic pathways of psychosocial health with weight gain is important. Public health policies and programmes should focus on educating pregnant women on the role of their lifestyle during pregnancy towards achieving healthy weight gain and subsequently provide support to women to achieve and maintain a healthy weight throughout their pregnancy. While sunlight exposure did not show an association with total gestational weight gain in this study, it remains an important modifiable factor in determining maternal and infant health outcomes, both for metabolic and psychosocial health, that warrants further investigation.

Future studies are needed to investigate the interaction of psychosocial health and environmental exposure in relation to total GWG. In addition, studies addressing other cofounding factors such as physical activity, sleep habits and nutritional intake would offer greater insights to the role of psychosocial health to gestational weight gain during pregnancy. It is important to highlight maternal modifiable lifestyle factors that may intervene with the programming of fetal health. While mental health and wellbeing is gaining importance in the modern society, it is crucial to study its long-lasting impact on the mother and the health of our future generations.

## Data Availability

The datasets generated and analysed during the current study are not publicly available due to ethical and privacy reasons but are available from the corresponding author on reasonable request.
